# Supplementary material for: Health care system efficiency and life expectancy: A 140-country study
Source: PLoS One. 2021 Jul 9;16(7):e0253450. doi: 10.1371/journal.pone.0253450 (PMC8270475; doi:10.1371/journal.pone.0253450)
Supplement: S1 Table — (DOCX) [file pone.0253450.s001.docx]

| **S1 Table. Definition of variables included in the analysis, from the Human Development Data (30).** | |
| --- | --- |
| Education Index | Average of mean years of schooling (of adults) and expected years of schooling (of children), both expressed as an index obtained by scaling with the corresponding maxima. |
| Infants lacking immunization DTP | Percentage of surviving infants who have not received their first dose of diphtheria, pertussis and tetanus vaccine. |
| Infants lacking immunization measles | Percentage of surviving infants who have not received the first dose of measles vaccine. |
| Health expenditure as percentage of the GDP | Spending on healthcare goods and services, expressed as a percentage of GDP. It excludes capital health expenditures such as buildings, machinery, information technology and stocks of vaccines for emergency or outbreaks. |
| Population using at least basic sanitation services | Percentage of the population using at least basic sanitation services, that is, improved sanitation facilities that are not shared with other households. This indicator encompasses people using basic sanitation services as well as those using safely managed sanitation services. Improved sanitation facilities include flush/pour flush toilets connected to piped sewer systems, septic tanks or pit latrines; pit latrines with slabs (including ventilated pit latrines); and composting toilets. |
| Unemployed in the labour force | Percentage of the labour force population ages 15 and older that is not in paid employment or self-employed but is available for work and has taken steps to seek paid employment or self-employment. |
| Income inequality, Gini coefficient | Measure of the deviation of the distribution of income among individuals or households within a country from a perfectly equal distribution. A value of 0 represents absolute equality, a value of 100 absolute inequality. |
| Old age dependency ratio | Ratio of the population ages 65 and older to the population ages 15–64, expressed as the number of dependants per 100 people of working age (ages 15–64). |
