# Supplementary material for: Health care system efficiency and life expectancy: A 140-country study
Source: PLoS One. 2021 Jul 9;16(7):e0253450. doi: 10.1371/journal.pone.0253450 (PMC8270475; doi:10.1371/journal.pone.0253450)
Supplement: S2 Table — (DOCX) [file pone.0253450.s002.docx]

| **S2 Table. List of countries included in the analysis and their estimated difference in life expectancy from the best practice line, given their level of health care expenditures; countries are listed alphabetically; the country number represents the code used on the efficiency frontier curve to identify the single countries in the Fig. 1.** | | |
| --- | --- | --- |
| **Numbe**r | **Country** | **Potential life expectancy improvement** |
| 1 | Afghanistan | 9.299 |
| 2 | Albania | 0.000 |
| 3 | Angola | 13.230 |
| 4 | Argentina | 4.068 |
| 5 | Armenia | 3.782 |
| 6 | Australia | 1.095 |
| 7 | Austria | 2.800 |
| 8 | Azerbaijan | 7.072 |
| 9 | Bangladesh | 0.000 |
| 10 | Belarus | 5.350 |
| 11 | Belgium | 3.000 |
| 12 | Belize | 2.449 |
| 13 | Benin | 9.998 |
| 14 | Bhutan | 4.092 |
| 15 | Bosnia and Herzegovina | 2.457 |
| 16 | Botswana | 10.472 |
| 17 | Brazil | 5.692 |
| 18 | Bulgaria | 5.705 |
| 19 | Burkina Faso | 11.795 |
| 20 | Burundi | 1.898 |
| 21 | Cabo Verde | 2.727 |
| 22 | Cambodia | 4.532 |
| 23 | Cameroon | 14.667 |
| 24 | Canada | 2.000 |
| 25 | Central African Republic | 8.109 |
| 26 | Chad | 18.423 |
| 27 | Chile | 1.593 |
| 28 | Colombia | 1.571 |
| 29 | Comoros | 9.535 |
| 30 | Congo | 10.428 |
| 31 | Congo (Democratic Republic of the) | 0.000 |
| 32 | Costa Rica | 0.000 |
| 33 | Croatia | 2.673 |
| 34 | Cyprus | 1.590 |
| 35 | Czechia | 3.292 |
| 36 | Denmark | 3.500 |
| 37 | Dominican Republic | 5.229 |
| 38 | Ecuador | 2.237 |
| 39 | Egypt | 5.188 |
| 40 | El Salvador | 4.181 |
| 41 | Estonia | 3.328 |
| 42 | Eswatini (Kingdom of) | 21.033 |
| 43 | Ethiopia | 2.131 |
| 44 | Finland | 2.397 |
| 45 | France | 1.800 |
| 46 | Gabon | 11.456 |
| 47 | Gambia | 6.158 |
| 48 | Georgia | 5.065 |
| 49 | Germany | 3.200 |
| 50 | Ghana | 9.856 |
| 51 | Greece | 0.048 |
| 52 | Guatemala | 2.775 |
| 53 | Guinea | 11.978 |
| 54 | Guinea-Bissau | 14.647 |
| 55 | Guyana | 5.297 |
| 56 | Haiti | 8.996 |
| 57 | Honduras | 0.834 |
| 58 | Hungary | 5.105 |
| 59 | Iceland | 1.267 |
| 60 | India | 4.844 |
| 61 | Indonesia | 4.161 |
| 62 | Iraq | 6.932 |
| 63 | Ireland | 2.500 |
| 64 | Israel | 0.000 |
| 65 | Italy | 0.178 |
| 66 | Jamaica | 2.563 |
| 67 | Japan | 0.000 |
| 68 | Jordan | 2.360 |
| 69 | Kazakhstan | 6.333 |
| 70 | Kenya | 7.042 |
| 71 | Korea (Republic of) | 0.263 |
| 72 | Kyrgyzstan | 2.521 |
| 73 | Lao People's Democratic Republic | 5.727 |
| 74 | Latvia | 5.529 |
| 75 | Lesotho | 21.784 |
| 76 | Liberia | 9.411 |
| 77 | Lithuania | 6.148 |
| 78 | Luxembourg | 2.300 |
| 79 | Madagascar | 5.922 |
| 80 | Malawi | 9.496 |
| 81 | Maldives | 2.227 |
| 82 | Mali | 12.093 |
| 83 | Malta | 1.270 |
| 84 | Mauritania | 8.575 |
| 85 | Mauritius | 4.986 |
| 86 | Mexico | 4.148 |
| 87 | Moldova (Republic of) | 5.381 |
| 88 | Mongolia | 6.975 |
| 89 | Mozambique | 8.113 |
| 90 | Namibia | 16.314 |
| 91 | Nepal | 2.964 |
| 92 | Netherlands | 2.200 |
| 93 | Nicaragua | 2.467 |
| 94 | Niger | 4.592 |
| 95 | Nigeria | 19.826 |
| 96 | Norway | 2.100 |
| 97 | Pakistan | 5.628 |
| 98 | Panama | 2.651 |
| 99 | Paraguay | 4.983 |
| 100 | Peru | 1.509 |
| 101 | Philippines | 4.148 |
| 102 | Poland | 2.838 |
| 103 | Portugal | 1.184 |
| 104 | Romania | 3.879 |
| 105 | Russian Federation | 8.234 |
| 106 | Rwanda | 4.468 |
| 107 | Saint Lucia | 1.713 |
| 108 | Sao Tome and Principe | 3.373 |
| 109 | Senegal | 5.886 |
| 110 | Serbia | 4.673 |
| 111 | Sierra Leone | 20.340 |
| 112 | Slovakia | 4.794 |
| 113 | Slovenia | 1.749 |
| 114 | South Africa | 15.900 |
| 115 | Spain | 0.000 |
| 116 | Sri Lanka | 0.000 |
| 117 | Sudan | 9.325 |
| 118 | Suriname | 7.054 |
| 119 | Sweden | 1.700 |
| 120 | Switzerland | 0.800 |
| 121 | Tajikistan | 2.910 |
| 122 | Tanzania (United Republic of) | 8.387 |
| 123 | Thailand | 0.829 |
| 124 | Timor-Leste | 6.052 |
| 125 | Togo | 11.826 |
| 126 | Trinidad and Tobago | 8.301 |
| 127 | Tunisia | 2.102 |
| 128 | Turkey | 2.300 |
| 129 | Turkmenistan | 11.460 |
| 130 | Uganda | 10.167 |
| 131 | Ukraine | 5.168 |
| 132 | United Kingdom | 2.748 |
| 133 | United States | 5.200 |
| 134 | Uruguay | 3.760 |
| 135 | Uzbekistan | 4.405 |
| 136 | Vanuatu | 2.075 |
| 137 | Venezuela (Bolivarian Republic of) | 4.650 |
| 138 | Viet Nam | 0.000 |
| 139 | Zambia | 10.431 |
| 140 | Zimbabwe | 13.533 |
