# Supplementary material for: Health care system efficiency and life expectancy: A 140-country study
Source: PLoS One. 2021 Jul 9;16(7):e0253450. doi: 10.1371/journal.pone.0253450 (PMC8270475; doi:10.1371/journal.pone.0253450)
Supplement: Attachment — Submitted filename: ResponseToReviewers.docx [file pone.0253450.s005.docx]

Dear Editor and dear Reviewers,

Thank you very much for you feedbacks, which we have addressed in the second revised version of the manuscript. Please find below our answers.

Reviewer #1: Dear Authors,
Thank you very much for your detailed explanation and modifications. I have some minor suggestions.

1. It's better to modify the topic to "Health care system efficiency and life …)

We have modified the title accordingly.

2. It is interesting to plot a correlation figure like you did in figure 1 using "efficiency score and change in life expectancy" for all countries.

We have produced the figure, called figure 2 in the text.

Reviewer #2: Thank you for addressing my second and third comments.
Thank you for providing the country data set.- I suggest some additional actions on this database:1
1. Provide the age-related data (missing on the CSV file)

We have no age-related data apart from the age dependency ratio, which is reported already in the file. We also don’t have age-specific data, as we are dealing with life expectancy at birth.

2. Separate the raw data (extracted from the UNDP) from calculated data points, such as the Efficiency index)

We have separated the raw data from the calculated data points: the new data file contains two data sheets, one for the raw data and one for the derived data.
